# Quantifying Adverse Childhood Experiences in Oklahoma With the Oklahoma Adversity Surveillance Index System (OASIS): Development and Cross-Sectional Study

**DOI:** 10.2196/45891

**Published:** 2023-08-22

**Authors:** Jason Walter Beaman, Cherie Josephine Miner, Cadence Bolinger

**Affiliations:** 1 School of Forensic Sciences Center for Health Sciences Oklahoma State University Tulsa, OK United States; 2 Department of Psychiatry and Behavioral Sciences Center for Health Sciences Oklahoma State University Tulsa, OK United States; 3 School of Community Health Sciences, Counseling and Counseling Psychology Oklahoma State University Stillwater, OK United States

**Keywords:** statewide intervention, adverse childhood experiences, public health, surveillance system, trauma, rural, mental illness, childhood trauma, surveillance, developmental trauma, adult

## Abstract

**Background:**

Developmental trauma depending on several factors may lead to later adult health risks and is an increasing public health concern, especially in states with predominantly rural populations. Oklahoma remains one of the states in America with the highest count of adverse childhood experiences (ACEs); therefore, more refined research methods for quantifying ACEs are vital for ensuring proper statewide interventions.

**Objective:**

While data sets already exist at the state level measuring specific ACEs like divorce or child abuse, the state currently lacks a single source for specific ACEs that can incorporate regions to allow for the identification of counties where ACEs are especially high. This county identification will allow for assessing trends in adversity prevalence over time to indicate where targeted interventions should be done and which counties experience amplified long-term consequences of high ACE rates. Thus, the model for the Oklahoma Adversity Surveillance Index System (OASIS) was born—a public health tool to map ACEs at the county level and grade them by severity over time.

**Methods:**

County-level data for 6 ACEs (mental illness, divorce, neglect, child abuse, domestic violence, and substance use) were collected from the Oklahoma Department of Human Services, Oklahoma State Department of Health, and Oklahoma Community Mental Health Centers for the years 2010 to 2018. First, a potential ACEs score (PAS) was created by standardizing and summing county rates for each ACE. To examine the temporal change in the PAS, a bivariate regression analysis was conducted. Additionally, an ACEs severity index (ASI) was created as a standardized measure of ACE severity across time. This included scoring counties based on severity for each ACE individually and summing the scores to generate an overall ASI for each county, capturing the severity of all ACEs included in the analysis.

**Results:**

Mental illness and substance use showed the highest rates at the state level. Results from the regression were significant (*F*_1,7_*_6_*=5.269; *P*=.02), showing that county PAS showed an increase over years. The ASI scores ranged from 0 to 6, and 4 Oklahoma counties (Adair, McCurtain, Muskogee, and Pittsburg) received a score of 6.

**Conclusions:**

OASIS involves the identification of counties where ACEs are most prevalent, allowing for the prioritization of interventions in these “hot spot” counties. In addition, regression analysis showed that ACEs increased in Oklahoma from 2010 to 2018. Future efforts should center on adding additional ACEs to the ASI and correlating adverse outcome rates (such as violence and medical disorder prevalence) at the county level with high ASI scores.

## Introduction

### Background

The Adverse Childhood Experiences Study, published in 1998, is a sentinel publication in modern health care [1]. This study demonstrated several important factors. First, childhood adversity is associated with poor outcomes as an adult, including cardiovascular disease, diabetes, obesity, liver and digestive diseases, and cancer [2-4]. Second, this correlation is dose-dependent—the more adversity, the higher the likelihood of poor outcomes [1]. Third, adversity in childhood extends beyond personal factors such as abuse and neglect and includes household dysfunction [1]. Since this study was first published, adverse childhood experiences (ACEs) have become common terminology in medicine [4,5]. With the strong correlation with adverse health outcomes, measuring the burden of ACEs can be important for public health and prevention. Secondary to the burden of disease and dysfunction, it is important to quantify the effect of ACEs on a community. Understanding this burden is essential for the deployment of limited resources and the implementation of evidence-based primary, secondary, and tertiary prevention programs.

A definitive list of ACEs does not exist. Those listed in the original Adverse Childhood Experiences Study questionnaire included physical, emotional, and sexual abuse; physical and emotional neglect; violence toward one’s mother; mental illness; substance abuse; and incarceration of a household member [1]. Other ACEs that should be considered according to the Behavioral Risk Factor Surveillance System (BRFSS) and Child Trends include educational and financial opportunities, chronic and infectious diseases, maternal health, risky behavior, injury, violence outside of the home, neighborhood safety, homelessness, bullying, racial and ethnic discrimination, and parent or guardian death [6,7].

Screening for childhood adversity and trauma is gaining acceptance and is recommended by the American Academy of Pediatrics [8]. It is unclear whether or not assessing ACEs at the patient level can alter health outcomes [9,10]. However, it has been demonstrated that patients are not averse to discussing the topic [11]. The more uptake there is in the use of patient instruments such as the ACEs questionnaire, the more useful this method can be in public health surveillance.

### Assessing ACEs Through Instruments

There are many different instruments available for assessing the number of ACEs an individual experiences. Most of these instruments will score the presence of an ACE in a dichotomous fashion, such as yes or no for the exposure. This method does not account for the intensity or duration of a particular traumatic experience. Further, this method does not allow for the experience to be evaluated in context. For example, a patient who is the child of a divorce may become separated from an abusive caregiver. Such circumstances underline the problems in attempting to understand and contextualize social dysfunction and the need for further development in methods for measuring and analyzing ACEs.

Perhaps the most common method of assessing ACEs is what was used in the Adverse Childhood Experiences Study, which was a retrospective survey [1]. This method can be used for public health programming and also for basic research. In the public health field, an ACE module was added to collect information on child abuse and neglect and household challenges through the BRFSS [12]. The ACE module was included at least once in the BRFSS of every state by the end of 2020 [13]. The BRFSS ACE module asks an individual to reflect on their childhood and answer a series of questions surrounding abuse, violence, drug use, and mental illness in the home which are adapted from the original Adverse Childhood Experiences Study [14]. Since 2009, the BRFSS has been collecting data on ACEs, and the module was updated in 2019 to include questions about neglect. Most recently, it was updated in 2021 with questions about childhood support and caregiver involvement.

Another important survey is the National Survey of Children’s Health (NSCH). This is an annual survey conducted in the United States that began in 2003 and has been reported as recently as 2021 [7,15]. One child under the age of 18 in each household is selected to be the focus of the survey. Specific ACE questions in the survey include parent or guardian divorce, separation, death, or incarceration; mental illness; suicide; illicit drug use; alcohol use; depression of a household member; domestic violence; neighborhood violence; income; and housing or food security [7]. In 2016, data collected by the NSCH indicated that 45% of children experienced at least one ACE, and 10% experienced 3 or more ACEs [7,16]. The most common ACEs reported were parental separation and low socioeconomic status [7].

Another method used to measure ACEs is by directly asking an individual as they intersect with the health care system [4,17]. This includes having the patient complete a screening instrument, such as the Adverse Childhood Experiences Questionnaire (ACE-Q), which can then be analyzed at a larger clinical level [11,18,19]. However, patient questionnaires also have shortcomings. First, they measure individuals presenting to a health care setting [18-20]. This is important because of well-known disparities in individuals accessing health care, including lack of a provider (such as in rural areas) or lack of ability to pay [21]. And while there is research indicating some patients feel comfortable discussing childhood trauma or ACEs with a health care provider [22], there are also patients with concerns about medical record storage in general, as issues with privacy arise in the increasingly digital world [23].

Knowledge of ACEs in a community could be important for primary, secondary, and tertiary prevention planning. Improved knowledge of prevalence rates could lead to larger gains in positive outcomes and more efficient use of precious resources. This is especially important in high-burden, low-resource areas, such as Oklahoma [24-27]. Oklahoma has consistently been recognized as leading the nation in childhood adversity, with 28.5% of Oklahoma children experiencing 2 or more ACEs [28]. Oklahoma also experiences poor social determinants of health (SDOH), such as health care shortages, violence, and food insecurity, among others [29]. Further, there is a lack of access to care due to a lack of primary care providers outside of metropolitan areas in rural communities, which make up the majority of Oklahoma [24-27]. These upstream deficiencies lead to high levels of adverse health outcomes such as diabetes, obesity, substance use disorders, and overdose mortality [24,30].

While it is known that Oklahomans have high rates of childhood adversity, there is no condensed source of data to which stakeholders can turn to receive aggregated, targeted information on ACEs at a granular (county) level. While there are great data sets like the BRFSS and NSCH, these lack county-level statistics for all of Oklahoma [31]. Currently, data is spread out across various state agencies, such as the Oklahoma State Courts Network (OSCN), the Oklahoma Department of Health Services (OKDHS), and Oklahoma community mental health centers (CMHCs). With multiple agencies doing different parts of the work in silos, Oklahoma does not have a coordinated trauma-response system in place to capture and publish all ACE data in one location.

To address this lack of a coordinated system and to combat this epidemic of childhood adversity and subsequent negative health consequences, the Oklahoma Adversity Surveillance Index System (OASIS) was created. OASIS was initiated in 2021 through a collaboration between the Oklahoma State University Center for Health Sciences and multiple state agencies. Data-sharing agreements were used to obtain data that were not publicly available. This system relies on several concepts. First, the adversity burden shared by a population should have a footprint in interaction with public agencies that collect or could collect data. Second, while there may be inaccuracies in data collection, these inaccuracies should be equally inaccurate over space and time. This allows for hot spot determination and comparisons among similar geographical entities (ie, county, zip code) and time (ie, years). Third, the data should be regularly collected and analyzed to monitor emerging areas and trends. Identifying trends can allow for the examination of intercounty differences and temporal variations between geographical entities. These trends can indicate where further analyses should be done to determine the factors that may contribute to the observed variations in ACE burden across counties and years. Such factors could include socioeconomic conditions, access to health care and support services, community resources, education levels, and public health initiatives. Finally, all efforts should be made to review and refine the system so that it becomes more accurate and insightful over time.

With the implementation of OASIS, the state of Oklahoma now has access to a public health tool that encapsulates county-level statewide data on ACEs that were previously unavailable or difficult to obtain. Additionally, with OASIS in place, the state will be able to carry out targeted interventions in the counties that need it the most. Without this surveillance system, high-burden counties would have been previously unidentifiable, and it would not have been possible to specifically target them with additional resources and prevention strategies. We hope that other states will replicate our methodology and identify their own hot spots to ultimately better serve areas that need more ACE-related support, resources, and services.

## Methods

### Overview

Data were obtained from various state agency sources for the years 2010 to 2018. Counts of abuse and neglect were obtained from the OKDHS. The OKDHS reports these data as a combination of physical, sexual, and psychological abuse, listed as a single “abuse” category. Ideally, these data would be separated, but currently, a county-level data set with these distinctions is unavailable. Neglect is listed separately. Data were available at the zip code and county level. OKDHS receives data from reports substantiated by child protective services (CPS). Data for divorce were obtained from the Oklahoma State Department of Health (OSDH). The OSDH collects divorce data for all counties in Oklahoma from county clerks. Unfortunately, Oklahoma does not inquire about whether or not children are involved when an individual files for divorce.

Data for parental mental illness and substance use were obtained at the zip code level for patients treated at Oklahoma CMHCs. CMHCs aggregate their data from appointments and patient files. This data are then submitted to the Oklahoma Department of Mental Health and Substance Abuse Services (ODMHSAS). Rates of mental illness and substance use were calculated at the population level. For divorce, mental illness, and substance use, information is not obtained as to whether an individual has children, so only raw numbers were used. Domestic violence rates were calculated through the number of charges filed as listed in the OSCN. Through the use of a publicly available application programming interface, records were searched at the county level. Again, this information may not be specific to parents. Data from the US Census Bureau were used to calculate population data for all counties. Counties were coded as metro or nonmetro following US Department of Agriculture Economic Research Service Rural Urban Continuum Codes.

### Potential ACE Score

To calculate additive totals, county crude rates for each ACE were standardized to 100 (average county crude rates per year are reported in the Results section). The available ACEs were added to create a potential ACE score (PAS). The PAS ranges from 0 to 600, representing the potential for children in each county to experience ACEs. Rates of determinants like divorce and substance use at the county level were used as proxies for the potential ACEs experienced by the children in that county.

### Statistical Analysis

Data were uploaded into SPSS (version 28.0.1.1; IBM Corp) for analysis. To examine the temporal changes in ACEs, a bivariate regression analysis was conducted. The PAS was used as the dependent variable, and the year was treated as the independent variable. This analysis allowed for the assessment of how the PAS changed over time. Results were visualized using Tableau (version 2021.4.5; Tableau Software, LLC).

### ACE Severity Index

In addition to the PAS, we developed the ACE severity index (ASI) as a standardized measure to assess the burden and severity of ACEs over time. The ASI aimed to identify counties more heavily impacted by ACEs, considering both the depth and duration of ACEs. To determine the impact of ACEs on specific counties, we scored each county based on ACE severity. Severity was measured for each ACE individually, assessing the extent of its occurrence over time. Counties were classified as “severe” for a particular ACE if its rate was above the state average. Severity scores ranged from 0 (indicating the county had an average or below-average rate for that ACE in all years) to 9 (indicating the county had an above-average rate for that ACE in all years). We calculated severity scores for each county for all 6 ACEs included in the analysis. Counties with severity scores above the average were given a point for that ACE, contributing toward their total ASI. This process was repeated for all 6 ACEs analyzed. The outcome was an ASI for each county, encompassing the severity of all ACEs included in the analysis and ranging from 0 to 6.

### Ethical Considerations

Data-sharing agreements were obtained before the project start. The project was approved as non–human subjects research by the institutional review board at Oklahoma State University Center for Health Sciences (#2021009). The requirement for informed consent was waived due to the retrospective nature and minimal risk of this study.

## Results

**Figure 1:**
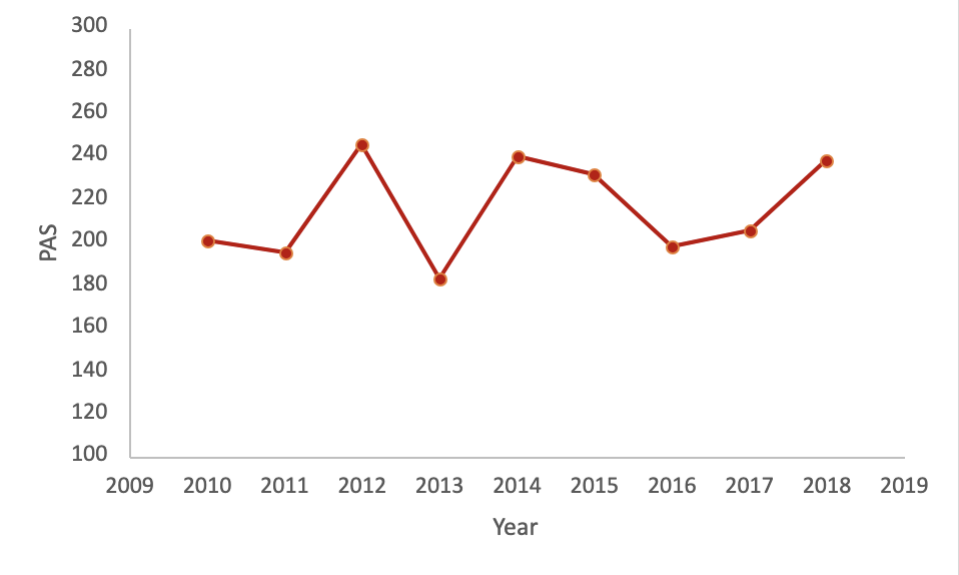
Yearly Oklahoma average PAS. PAS: potential adverse childhood event score.

Over the study period (2010-2018), the average PAS was 215.08, with an overall increasing trend from 2010 to 2018 (Figure 1). An analysis of the data set revealed notable trends in several Oklahoma counties regarding their PAS from 2010 to 2018. Specifically, Choctaw and Greer counties showed an increasing trend over the study period, while Cimarron and Harper counties consistently remained below the average PAS. Adair, Woodward, Coal, Muskogee, Pittsburg, and Brian counties consistently maintained a PAS above the average.

Choctaw County exhibited a consistent upward trend in PAS over the years, with a score of 274.637 in 2010 and 322.841 in 2011, reaching a peak of 439.225 in 2012, and still maintaining a score in the top 10 of counties in 2018 (335.35). Greer County also experienced an increasing trend, starting at 171.454 in 2010 and steadily rising to 302.128 in 2018. On the other hand, Cimarron County consistently remained below the average PAS throughout the study period. The county had a relatively low PAS of 83.137 in 2010 and showed slight fluctuations over the years, but remained consistently below average, reaching 108.26 in 2018. Similarly, Harper County started with a PAS of 163.285 in 2010 and remained consistently below average, with a score of 114.229 in 2018.

In contrast, Adair County consistently displayed remarkable PAS well above the average throughout the study period. Starting with a high PAS of 408.354 in 2010, Adair County maintained consistently elevated scores, reaching its peak of 433.736 in 2018. Similarly, Woodward County demonstrated a pattern of consistently scoring above the average. Beginning at 295.013 in 2010, Woodward County steadily increased its PAS over the years, culminating in a peak of 369.031 in 2018. Joining them in consistently exhibiting higher-than-average PAS were several other notable counties. Coal County, despite experiencing some fluctuations, maintained relatively high scores throughout the study period, with its highest PAS of 404.271 recorded in 2010 and remaining significantly above average at 309.158 in 2018. Muskogee County followed a similar trajectory, with a starting PAS of 277.018 in 2010 and a peak of 322.252 in 2018. Pittsburg County consistently scored above the average as well, reaching a peak PAS of 319.14 in 2018. Notably, Bryan County had a consistent above-average pattern, starting at 281.216 in 2010 and reaching its peak of 242.676 in 2018. These counties, including Adair and Woodward, stood out for their remarkable and consistent above-average PAS throughout the study period. Notably, the top 10 counties in 2018 had higher PAS values compared to the top ten counties in 2010 (Table 1).

**Table 1:** Top 10 Oklahoma counties for PAS^a^ in 2010 and 2018.

Ranking	2010		2018	
	County	PAS	County	PAS
1	Adair	408.354	Adair	433.74
2	Coal	404.271	Woodward	369.03
3	Beckham	331.141	Kay	347.2
4	Marshall	307.987	Seminole	345.48
5	Seminole	304.522	Choctaw	335.35
6	Woodward	295.013	Muskogee	322.25
7	Okfuskee	283.525	Pittsburg	319.14
8	Bryan	281.216	Ottawa	319.08
9	Muskogee	277.018	Blaine	316.2
10	Pittsburg	275.589	Latimer	312.23

^a^PAS: potential adverse childhood event score.

Our analysis revealed a significant relationship between year and PAS (*F*_1,76_=5.269; *P*=.02) (Table 2). For each 1 unit increase in the year variable, there was a corresponding 2.193 increase in the PAS (SE 0.955, *t*_1,76_=2.295; *P*=.02). The model summary indicates that the regression model explains a small proportion of the variance in the PAS.

**Table 2:** Model summary of regression analysis assessing the relationship between year and potential adverse childhood event score. The dependent variable was potential adverse childhood event score.

Model	*R*	*R* ^2^	Adjusted *R*^2^	Estimate SE	Change in statistics		
					Change in *R*^2^	Change in *F* (*df*)	*P* value
1	0.087^a^	0.008	0.006	64.94422332	0.008	5.269 (1,691)	.02

^a^Predictors were (constant), year.

**Figure 2:**
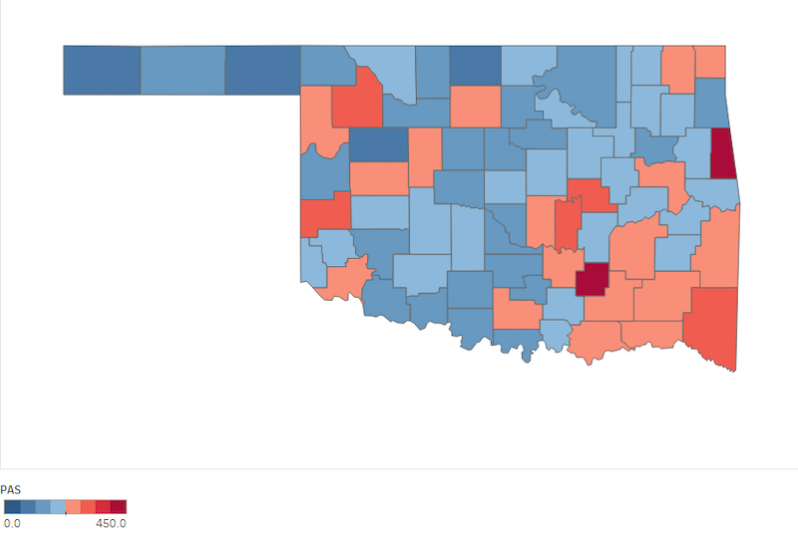
Oklahoma PAS county distribution in 2010. PAS: potential adverse childhood event score.

**Figure 3:**
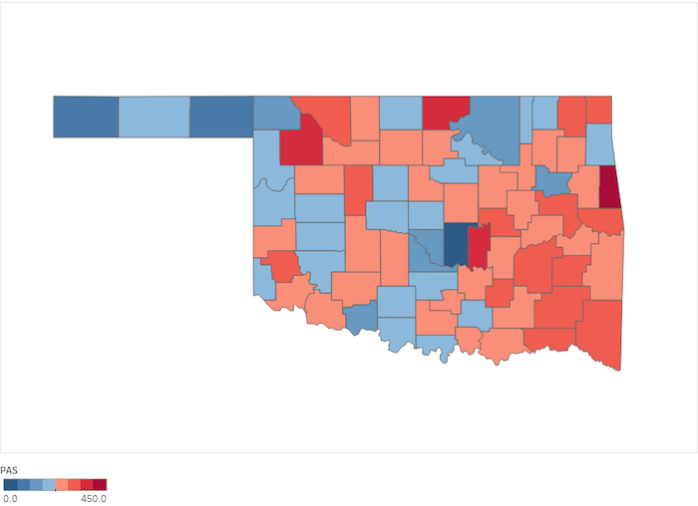
Oklahoma PAS county distribution in 2018. PAS: potential adverse childhood event score.

When visualized, the difference between county PAS in 2010 and 2018 shows an overall increase (Figures 2 and 3).

**Figure 4:**
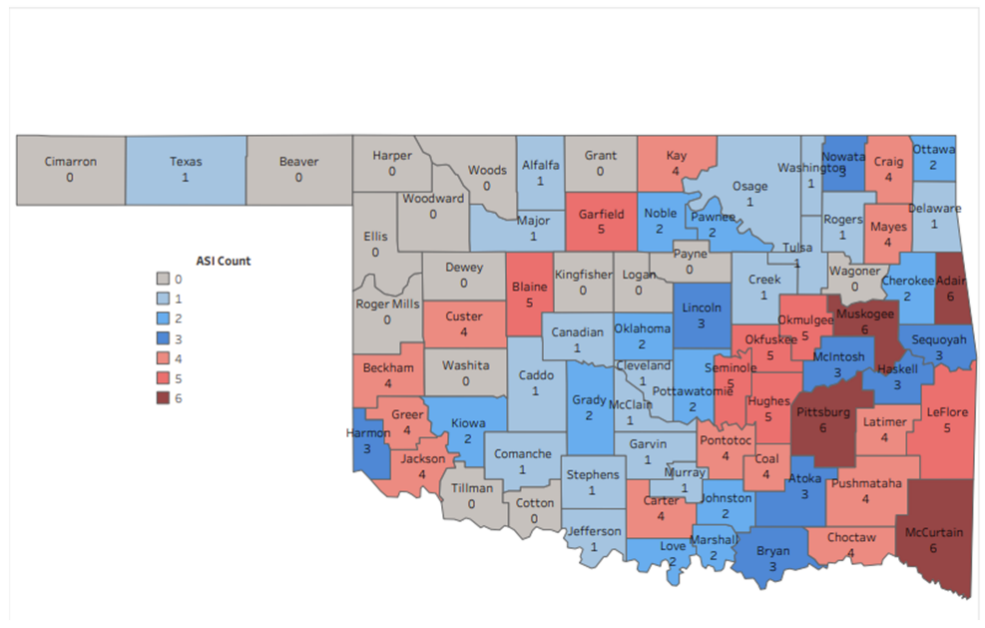
Map of Oklahoma Adversity Surveillance Index System (OASIS) showing the ASI for each Oklahoma county. Zero represents the lowest burden and 6 the highest burden encompassing all 6 adverse childhood events included in our analysis. ASI: adverse childhood event severity index.

The results for the ASI showed that 4 nonmetro Oklahoma counties—Adair, McCurtain, Muskogee, and Pittsburg—had scores of 6 (Figure 4). Garfield, Blaine, Seminole, Okfuskee, Okmulgee, Hughes, and LeFlore counties received scores of 5.

## Discussion

### Principal Findings

In this study, we aimed to examine the prevalence of ACEs and their variations across counties in Oklahoma using OASIS. Our findings provide valuable insights into the burden of ACEs in the state and highlight the need for targeted interventions and resource allocation. The analysis of the PAS revealed an increasing trend in ACE burden from 2010 to 2018, and our regression analysis demonstrated a significant relationship between the year and PAS, indicating a continuous rise in ACE burden over time.

The ASI allowed us to identify counties more heavily impacted by ACEs. After indexing counties on the ASI, 4 counties stood out, with scores of 6 of 6. All 4 counties are rural, nonmetro, located in Indian Country, are significantly below the national median income, have a greater percentage of persons living in poverty, and have higher unemployment rates than the national average [32]. All of these poor SDOH are known upstream risk factors for ACEs [7]. Much more in-depth analysis of these counties, along with others with high ASI scores, should be done to ascertain how their poor SDOH are driving childhood adversity.

The average county in Oklahoma exhibited rates of ACEs that were consistently above the state average for 4 or more years. This increasing trend is concerning and shows the need for further expansion of OASIS with the addition of other ACEs, for example, incarceration of a family member; Oklahoma consistently ranks in the top 10 for incarceration rate in the United States over the past decade [33]. Our analysis of the PAS showed that there was a significant relationship between year and PAS (*F*_1,691_=5.269; *P*=.02). For every 1 unit increase by year, there was a 2.193 (SE 0.955) unit increase in PAS (*t*_1,76_=2.295; *P*=.02).

OASIS has several strengths and limitations. The strengths include the creation of a framework that can be built upon and improved over time. With OASIS, we have designed and implemented a framework for data sharing and analysis across state data sets, an accomplishment that is not necessarily easy to do given the sensitivity around information on ACE variables. Having created a proof of concept, legislators and agency heads can understand how the way they collect data intersects with other agencies and allows judicious spending of resources. Another strength is the ability to compare geographical entities together. Comparing zip codes in large urban areas could be valuable to understanding population disparities, as survey studies of ACE prevalence have known weaknesses [34]. To generalize the results of a survey to the general population, it should be administered to a sufficiently large and randomized sample of the general population. This can be difficult to achieve. For example, the population mixture of the original Adverse Childhood Events Study was primarily White, health-insured individuals [35]. The BRFSS has similar concerns. Analysis has shown that individuals answering the ACEs module are more likely to have higher education than the US average [6,35]. These differences are notable in health care because it is well documented that there are significant health disparities among races, insured and uninsured people, and different levels of education [36-40]. Thus, with OASIS, the state of Oklahoma now has a way to easily access county-level ACE-related data that are generalizable to the whole state. This public health tool allows stakeholders to better identify areas that need more ACE-related support, resources, and services. Understanding why is the first step toward prevention.

Another strength is the ability to identify hot spots by depth and time. The PAS allows for counties to be compared across time to see if their rates of childhood adversity are increasing or decreasing. In addition, the PAS allows for counties to be compared to each other and trends identified between counties. We found that some counties, such as Adair, ranked consistently higher on the PAS compared to other counties. On the other hand, some counties, such as Harper and Cimarron, consistently ranked below the average PAS. These counties showing lower rates over time should be studied further; they can serve as models for successful prevention strategies that could be replicated in other areas.

OASIS is not without limitations, however. First is the lack of data on all known ACEs. For example, at one time, Oklahoma was the second most prevalent place in the world to incarcerate women and incarcerated more men and women combined than any other state in the United States [41,42]. Yet the Oklahoma Department of Corrections does not systematically collect data on inmates and their children. This prevents the understanding of this trauma as it relates to other ACEs and also prevents understanding the damage mass female incarceration can have on a community. One additional limitation of the surveillance system is the absence of validity testing, which means that the extent of error or inaccuracy within the system is unknown. Consequently, there is a risk of incorrectly identifying hot spots. However, it should be noted that since all data were collected using the same method, any inaccuracies should be consistent across different areas. Nonetheless, it is crucial for future efforts to develop methods that can test the validity of the system and address its identified weaknesses. By doing so, improvements can be made to enhance accuracy and ensure more reliable identification of hot spots. Another weakness lies in the extrapolation of population data without accounting for the number of children involved. The collected data for divorce, for example, are based on raw divorce numbers, without considering divorces that specifically affect children, as that information is not available from ODMHSAS court records. Consequently, there is a possibility of overcounting divorces involving children. However, it is important to note that this overcounting issue is consistent across all counties, as the same calculation method is used. Therefore, while the accuracy may be compromised, it is uniformly inaccurate across all counties.

A final limitation are the data themselves. OASIS relies on individuals accessing services and does not include those that do not. For example, if a mother accessed mental health treatment outside of a CMHC, that would not be measured. This is less of a problem in rural areas that have limited providers. However, in the urban areas of Oklahoma City and Tulsa, this likely leads to undercounting. This limitation is highlighted again in divorce. Individuals do not have to be married to raise children together. Therefore, when unmarried parents separate, that would not be reflected in OSDH divorce data, and this would lead to an undercounting of events.

### Conclusions

The findings of our study have significant implications for the field of public health and can shape future interventions and strategies. The identification of counties with a high prevalence of ACEs through OASIS allows for targeted interventions and prioritization of resources in these hot spot areas. Tailored interventions at the county level are crucial, considering the variations in ACE burden across different regions.

Our study highlights the value of the ASI in revealing geographic disparities in the severity and persistence of ACEs. Four counties in particular stood out, with an ASI score of 6, indicating an elevated prevalence of ACEs and consistently above-average rates across all ACE types examined. This signifies a pressing need for urgent and coordinated action from policymakers, health practitioners, social care providers, and researchers in addressing this public health issue. Counties with high ASI scores are likely to experience amplified long-term consequences associated with ACEs, including increased risks for chronic diseases, reduced educational achievement, and a higher likelihood of involvement with the criminal justice system [43,44]. It is imperative to respond promptly with both ACE prevention and effective management strategies to mitigate these outcomes. The ASI also serves as a valuable guide for targeted resource allocation. By identifying counties burdened by ACEs, resources can be efficiently prioritized, focusing on services such as trauma-informed care, community health initiatives, and educational interventions tailored to address ACEs. Future research should explore community-level factors (eg, poverty, community violence, service accessibility) as well as individual and family-level dynamics to design effective prevention and intervention strategies.

While acknowledging the limitations of this study, OASIS provides a foundation that can be refined and improved over time. This surveillance system facilitates collaboration and coordination across sectors, states, and agencies, enabling a comprehensive response to high-need areas. Future efforts should focus on expanding the range of ACEs, improving data collection, adding adverse outcome rates, and conducting validity testing. The ultimate objective of OASIS should be to develop effective strategies that not only address the present situation in counties with high childhood adversity indicated by the ASI but also prevent future generations from experiencing similar levels of adversity.

## Abbreviations

ACE-Q: Adverse Childhood Experiences Questionnaire
ACE: adverse childhood experience
ASI: adverse childhood experience severity index
BRFSS: Behavioral Risk Factor Surveillance System
CMHC: community mental health center
CPS: child protective services
NSCH: National Survey of Children’s Health
OASIS: Oklahoma Adversity Surveillance Index System
ODMHSAS: Oklahoma Department of Mental Health and Substance Abuse Services
OKDHS: Oklahoma Department of Human Services
OSDH: Oklahoma State Department of Health
PAS: potential adverse childhood experience score
SDOH: social determinants of health

## Data Availability

The data sets generated during and/or analyzed during the current study are available from the corresponding author upon reasonable request.
